# Crystal structures of two *ansa*-titanocene tri­fluoro­methane­sulfonate complexes bearing the Me_2_Si(C_5_Me_4_)_2_ ligand

**DOI:** 10.1107/S2056989016018363

**Published:** 2016-11-22

**Authors:** Monty Kessler, Christian Godemann, Anke Spannenberg, Torsten Beweries

**Affiliations:** a Leibniz-Institut für Katalyse e. V. an der Universität Rostock, Albert-Einstein-Strasse 29a, 18059 Rostock, Germany

**Keywords:** crystal structure, titanium metallocene, tri­fluoro­methane­sulfonate ligand

## Abstract

The crystal structures of two *ansa*-titanocene tri­fluoro­methane­sulfonate complexes bearing the Me_2_Si(C_5_Me_4_)_2_ ligand are reported, namely [di­methylbis­(η^5^-tetra­methyl­cyclo­penta­dien­yl)silane](tri­fluoro­methane­sulfonato-κ^2^ *O*,*O*′)titanium(III) toluene monosolvate, [Ti(CF_3_O_3_S)(C_20_H_30_Si)]·C_7_H_8_, **1**, and chlorido­[di­methyl­bis­(η^5^-tetra­methyl­cyclo­penta­dien­yl)silane](tri­fluoro­methane­sulfonato-κ*O*)titanium(IV), [Ti(CF_3_O_3_S)(C_20_H_30_Si)Cl], **2**. Both complexes display a bent metallocene unit, the metal atom being coordinated in a distorted tetra­hedral geometry, with the tri­fluoro­methane­sulfonate anion acting as a bidentate or monodentate ligand in **1** and **2**, respectively. In **1**, weak π–π stacking inter­actions involving the toluene solvent mol­ecules [centroid-to-centroid distance = 3.9491 (11) Å] are observed.

## Abstract 2

The crystal structures of two *ansa*-titanocene tri­fluoro­methane­sulfonate complexes bearing the Me_2_Si(C_5_Me_4_)_2_ ligand are reported. Both complexes display a bent metallocene unit, the metal centre is coordinated in a distorted tetra­hedral geometry.

## Chemical context   

Titanocene tri­fluoro­methane­sulfonate complexes have been investigated by our group as model complexes for overall water splitting (Kessler *et al.*, 2011 ▸; Hollmann *et al.*, 2013 ▸; Godemann *et al.*, 2015 ▸). We have found that the nature of the cyclo­penta­dienyl ligands strongly influences the outcome of the reaction. In case of the unbridged Ti^III^ complex Cp*_2_Ti(OTf) (**A**, Cp* = η^5^-penta­methyl­cyclo­penta­dien­yl), reaction with water gave di­hydrogen and the Ti^IV^ complex Cp*_2_Ti(OH)(OTf), which could not be reconverted photochemically into the Ti^III^ starting material (Kessler *et al.*, 2011 ▸; Hollmann *et al.*, 2013 ▸). In contrast, reaction of the silanediyl-bridged complex Me_4_Si_2_(C_5_Me_4_)_2_Ti(OTf) (**B**) with water was found to yield the Ti^III^ compound [Me_4_Si_2_(C_5_Me_4_)Ti(H_2_O)_2_](OTf), which could be oxidized with TEMPO to give a Ti^IV^ species [Me_4_Si_2_(C_5_Me_4_)_2_Ti(H_2_O)(OH)](OTf). Photolysis of the latter results in a photoreduction and elimination of the OH ligand to give a Ti^III^ tri­fluoro­methane­sulfonate complex. Several cycles of this synthetic model scheme for water splitting can be passed (Godemann *et al.*, 2015 ▸).
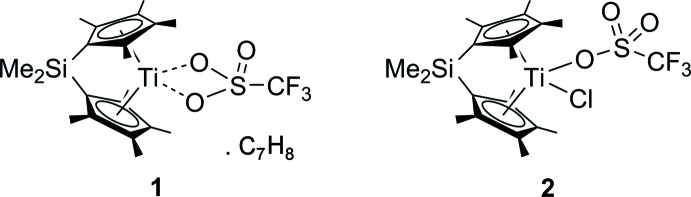


Variation of the *ansa*-cyclo­penta­dienyl ligand, *i.e.* shortening of the bridging unit, should have an influence on the reactivity of the corresponding tri­fluoro­methane­sulfonate complexes. We therefore aimed at the synthesis of bridged tri­fluoro­methane­sulfonate complexes bearing the metallocene [Me_2_Si(C_5_Me_4_)Ti].

## Structural commentary   

**Figure 1:**
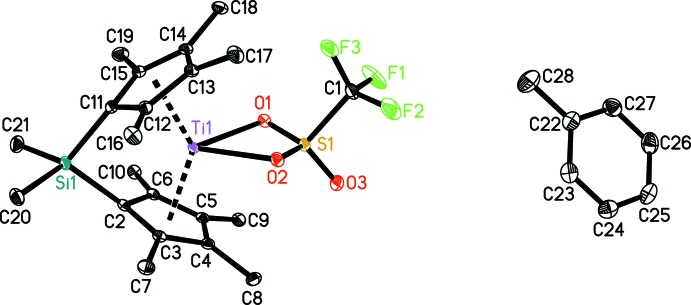
The structures of the molecular components of compound **1**. Displacement ellipsoids correspond to the 30% probability level. H atoms have been omitted for clarity.

Figs. 1 ▸ and 2 ▸ show displacement ellipsoid plots of [Me_2_Si(C_5_Me_4_)_2_Ti(OTf)]·C_7_H_8_ (**1**) and [Me_2_Si(C_5_Me_4_)_2_Ti(OTf)Cl] (**2**), respectively. Both metal complexes exhibit distorted tetra­hedral coordination geometries and show the typical bent metallocene moiety.

Complex **1** crystallizes with one mol­ecule of toluene in the asymmetric unit. The crystal structure of **1** confirms the bidentate binding mode of the tri­fluoro­methane­sulfonate ligand, which is in contrast to other complexes bearing different metallocene units (Kessler *et al.*, 2011 ▸; Godemann *et al.*, 2015 ▸).

However, this binding mode is known for group 4 complexes (Giannini *et al.*, 1997 ▸; Donkervoort *et al.*, 1997 ▸; Basuli, Bailey *et al.*, 2003 ▸; Basuli, Huffman, & Mindiola, 2003 ▸; Basuli *et al.*, 2004 ▸). Metallocene compounds such as the lanthanide compounds [C_5_H_3_(SiMe_3_)_2_]_2_Nd(*κO*-OTf)(*κ* ^2^ *O,O*-OTf) (Hitchcock *et al.*, 2006 ▸) and [C_5_H_2_(*t*-Bu)_3_]_2_Ce(*κ* ^2^ *O,O*-OTf) (Werkema *et al.*, 2013 ▸) have been reported as well. Compared to the above mentioned titanocene tri­fluoro­methane­sulfonate, which shows the tri­fluoro­methane­sulfonate ligand in *κ* ^1^ coord­ination, the Ti—O bonds are significantly longer in the title compound **1**, pointing towards a much weaker coordination of the ligand in a symmetrical arrangement [for comparison: **A**: 2.078 (1), **B**: 2.058 (2) Å].

Titanocene(IV) complexes with a tri­fluoro­methane­sulfonate ligand in a κ^1^-binding mode have been described by Beckhaus *et al.* (1994 ▸); Taw *et al.* (2003 ▸); Deacon *et al.* (2006 ▸); Kessler *et al.* (2011 ▸) and Godemann *et al.* (2015 ▸). The crystal structure of complex **2** also shows the tri­fluoro­methane­sulfonate ligand in a κ^1^-binding mode with a Ti1—O1 distance of 2.0605 (11) Å, which is slightly shorter compared to the bis­(penta­methyl­cyclo­penta­dien­yl) compound Cp*_2_Ti(Cl)(OTf) [2.097 (4) Å; Beckhaus *et al.*, 1994 ▸]. The value for the Ti1—Cl1 bond length [2.3255 (5) Å] is in the expected range for a Ti^IV^–chloride bond and is the same as found for the above Cp* complex [2.328 (2) Å].

## Supra­molecular features   

For **1**, weak π–π stacking inter­actions were observed between two neighbouring toluene solvent mol­ecules along the *a* axis [distance between ring centroids 3.9491 (11) Å and ring slippage of 1.985 Å].

## Synthesis and crystallization   

All operations were carried out under argon with standard Schlenk techniques or in a glovebox. The alkyne complex Me_2_Si(C_5_Me_4_)_2_Ti(η^2^-Me_3_SiC_2_SiMe_3_) was prepared according to a published procedure (Varga *et al.*, 1997 ▸). Yb(OTf)_3_ was purchased from Sigma Aldrich and used as received. Toluene was purified with the Grubbs-type column system ‘Pure Solv MD-5’ and dispensed into thick-walled glass Schlenk bombs equipped with Young-type Teflon valve stopcocks.

**Synthesis of 1:** Me_2_Si(C_5_Me_4_)_2_Ti(η^2^-Me_3_SiC_2_SiMe_3_) (0.450 g, 0.87 mmol) and Yb(OTf)_3_ (0.730 g, 1.17 mmol) were dissolved in 30 ml of toluene and heated at 333 K overnight, resulting in a colour change from dark yellow to green. All volatiles were removed *in vacuo* and the residue was again dissolved in toluene. The solution was filtered and the solvent was evaporated in vacuum to yield complex **1** as a dark-green powder. Single crystals suitable for an X-ray analysis were obtained from a saturated toluene solution at 195 K.

**Synthesis of 2:** In an experiment which aimed at the synthesis of the above Ti^III^ tri­fluoro­methane­sulfonate complex **1**, a batch of the alkyne complex Me_2_Si(C_5_Me_4_)_2_Ti(η^2^-Me_3_SiC_2_SiMe_3_) was used that contained significant amounts of the monochloride complex Me_2_Si(C_5_Me_4_)_2_TiCl, which was formed by incomplete reduction of the dichloride complex Me_2_Si(C_5_Me_4_)_2_TiCl_2_ during synthesis of the alkyne complex. Reaction of the monochloride complex with Yb(OTf)_3_ yields the *ansa*-titanocene(IV) chloride tri­fluoro­methane­sulfonate complex **2**. Single crystals suitable for an X-ray analysis were obtained from a saturated toluene solution by slow cooling from 353 K to room temperature.

## Refinement   

Crystal data, data collection and structure refinement details are summarized in Table 1 ▸. All H atoms were placed geom­etrically and refined using a riding-atom approximation, with C—H = 0.95–0.98 Å, and with *U*_iso_(H) = 1.2*U*_eq_(C) or 1.5*U*_eq_(C) for methyl H atoms. A rotating model was used for the methyl groups.

## Supplementary Material

Crystal structure: contains datablock(s) 1, 2, New_Global_Publ_Block. DOI: 10.1107/S2056989016018363/rz5199sup1.cif

Structure factors: contains datablock(s) 1. DOI: 10.1107/S2056989016018363/rz51991sup2.hkl

Structure factors: contains datablock(s) 2. DOI: 10.1107/S2056989016018363/rz51992sup3.hkl

CCDC references: 1517524, 1517523

Additional supporting information:  crystallographic information; 3D view; checkCIF report

## Figures and Tables

**Figure 2:**
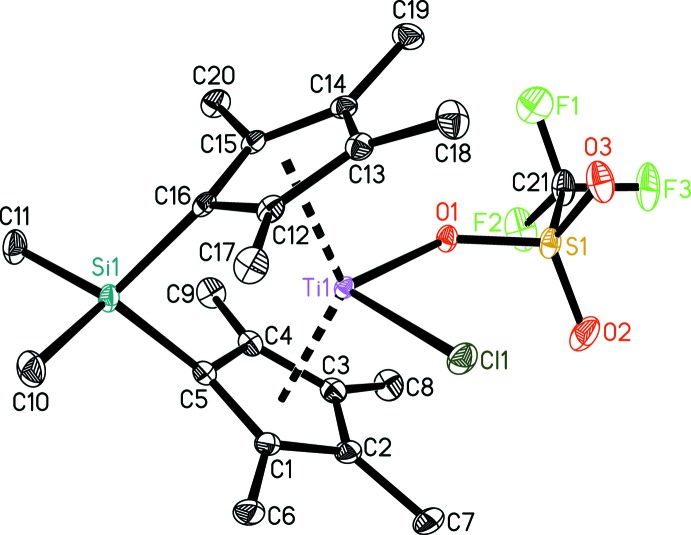
The mol­ecular structure of complex **2**. Displacement ellipsoids correspond to the 30% probability level. H atoms have been omitted for clarity.

**Table 1:** Experimental details

	**1**	**2**
Crystal data		
Chemical formula	[Ti(CF_3_O_3_S)(C_20_H_30_Si)]·C_7_H_8_	[Ti(CF_3_O_3_S)(C_20_H_30_Si)Cl]
*M*_r_	587.63	530.95
Crystal system, space group	Triclinic, *P* 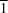	Monoclinic, *P*2_1_/*n*
Temperature (K)	150	150
*a*, *b*, *c* (Å)	8.9431 (2), 12.3682 (3), 13.8860 (3)	10.0958 (3), 15.8656 (5), 14.5544 (5)
α, β, γ (°)	66.795 (1), 85.501 (1), 86.367 (1)	90, 91.9841 (8), 90
*V* (Å^3^)	1406.44 (6)	2329.87 (13)
*Z*	2	4
Radiation type	Mo *K*α	Mo *K*α
μ (mm^−1^)	0.47	0.67
Crystal size (mm)	0.52 × 0.32 × 0.32	0.46 × 0.39 × 0.27

Data collection		
Diffractometer	Bruker APEXII CCD	Bruker APEXII CCD
Absorption correction	Multi-scan (*SADABS*; Bruker, 2014 ▸)	Multi-scan (*SADABS*; Bruker, 2014 ▸)
*T*_min_, *T*_max_	0.79, 0.86	0.78, 0.84
No. of measured, independent and observed [*I* > 2σ(*I*)] reflections	45127, 6782, 6191	34539, 5616, 5121
*R*_int_	0.022	0.021
(sin θ/λ)_max_ (Å^−1^)	0.661	0.661

Refinement		
*R*[*F* ^2^ > 2σ(*F* ^2^)], *wR*(*F* ^2^), *S*	0.030, 0.084, 1.04	0.030, 0.085, 1.04
No. of reflections	6782	5616
No. of parameters	345	290
H-atom treatment	H-atom parameters constrained	H-atom parameters constrained
Δρ_max_, Δρ_min_ (e Å^−3^)	0.53, −0.44	0.52, −0.43

Computer programs: *APEX2* (Bruker, 2014 ▸), *SAINT* (Bruker, 2013 ▸), *SHELXS97* and *XP* in *SHELXTL* (Sheldrick, 2008 ▸), *SHELXL2014* (Sheldrick, 2015 ▸), *publCIF* (Westrip, 2010 ▸) and *PLATON* (Spek, 2009 ▸).

## supplementary crystallographic information

### (1) [Dimethylbis(η5-tetramethylcyclopentadienyl)silane](trifluoromethanesulfonato-κ2O,O')titanium(III) toluene monosolvate Crystal data

**Table:**

[Ti(CF_3_O_3_S)(C_20_H_30_Si)]·C_7_H_8_	*Z* = 2
*M**_r_* = 587.63	*F*(000) = 618
Triclinic, *P*1	*D*_x_ = 1.388 Mg m^−^^3^
*a* = 8.9431 (2) Å	Mo *K*α radiation, λ = 0.71073 Å
*b* = 12.3682 (3) Å	Cell parameters from 9883 reflections
*c* = 13.8860 (3) Å	θ = 2.3–28.8°
α = 66.795 (1)°	µ = 0.47 mm^−^^1^
β = 85.501 (1)°	*T* = 150 K
γ = 86.367 (1)°	Prism, green
*V* = 1406.44 (6) Å^3^	0.52 × 0.32 × 0.32 mm

### (1) [Dimethylbis(η5-tetramethylcyclopentadienyl)silane](trifluoromethanesulfonato-κ2O,O')titanium(III) toluene monosolvate Data collection

**Table:**

Bruker APEXII CCD diffractometer	6782 independent reflections
Radiation source: fine-focus sealed tube	6191 reflections with *I* > 2σ(*I*)
Curved graphite monochromator	*R*_int_ = 0.022
φ and ω scans	θ_max_ = 28.0°, θ_min_ = 1.8°
Absorption correction: multi-scan (SADABS; Bruker, 2014)	*h* = −11→11
*T*_min_ = 0.79, *T*_max_ = 0.86	*k* = −16→16
45127 measured reflections	*l* = −17→18

### (1) [Dimethylbis(η5-tetramethylcyclopentadienyl)silane](trifluoromethanesulfonato-κ2O,O')titanium(III) toluene monosolvate Refinement

**Table:**

Refinement on *F*^2^	0 restraints
Least-squares matrix: full	Hydrogen site location: inferred from neighbouring sites
*R*[*F*^2^ > 2σ(*F*^2^)] = 0.030	H-atom parameters constrained
*wR*(*F*^2^) = 0.084	*w* = 1/[σ^2^(*F*_o_^2^) + (0.0418*P*)^2^ + 0.821*P*] where *P* = (*F*_o_^2^ + 2*F*_c_^2^)/3
*S* = 1.04	(Δ/σ)_max_ = 0.001
6782 reflections	Δρ_max_ = 0.53 e Å^−^^3^
345 parameters	Δρ_min_ = −0.44 e Å^−^^3^

### (1) [Dimethylbis(η5-tetramethylcyclopentadienyl)silane](trifluoromethanesulfonato-κ2O,O')titanium(III) toluene monosolvate Special details

**Table:**

Geometry. All esds (except the esd in the dihedral angle between two l.s. planes) are estimated using the full covariance matrix. The cell esds are taken into account individually in the estimation of esds in distances, angles and torsion angles; correlations between esds in cell parameters are only used when they are defined by crystal symmetry. An approximate (isotropic) treatment of cell esds is used for estimating esds involving l.s. planes.

### (1) [Dimethylbis(η5-tetramethylcyclopentadienyl)silane](trifluoromethanesulfonato-κ2O,O')titanium(III) toluene monosolvate Fractional atomic coordinates and isotropic or equivalent isotropic displacement parameters (Å^2^)

**Table:**

	*x*	*y*	*z*	*U*_iso_*/*U*_eq_	
C1	0.58968 (16)	0.74786 (14)	0.53570 (12)	0.0259 (3)	
C2	1.23125 (13)	0.84024 (11)	0.28241 (10)	0.0159 (2)	
C3	1.20796 (14)	0.71780 (11)	0.34743 (10)	0.0172 (2)	
C4	1.17246 (14)	0.70731 (11)	0.45198 (10)	0.0171 (2)	
C5	1.16963 (14)	0.82084 (11)	0.45390 (10)	0.0164 (2)	
C6	1.20392 (13)	0.90340 (11)	0.35053 (10)	0.0162 (2)	
C7	1.24135 (17)	0.61244 (12)	0.31968 (12)	0.0242 (3)	
H7A	1.3388	0.5763	0.3454	0.036*	
H7B	1.2442	0.6373	0.2433	0.036*	
H7C	1.1629	0.5551	0.3523	0.036*	
C8	1.15648 (16)	0.59327 (12)	0.54552 (11)	0.0231 (3)	
H8A	1.2562	0.5580	0.5662	0.035*	
H8B	1.0995	0.5393	0.5276	0.035*	
H8C	1.1033	0.6080	0.6039	0.035*	
C9	1.15069 (15)	0.84959 (13)	0.54951 (11)	0.0215 (3)	
H9A	1.0986	0.7860	0.6066	0.032*	
H9B	1.0915	0.9234	0.5334	0.032*	
H9C	1.2495	0.8581	0.5711	0.032*	
C10	1.23222 (16)	1.03016 (12)	0.32731 (11)	0.0211 (3)	
H10A	1.1439	1.0660	0.3511	0.032*	
H10B	1.2518	1.0727	0.2516	0.032*	
H10C	1.3194	1.0344	0.3641	0.032*	
C11	1.00179 (14)	0.90793 (11)	0.15095 (10)	0.0174 (2)	
C12	0.91352 (14)	0.80494 (12)	0.18039 (10)	0.0185 (2)	
C13	0.77183 (14)	0.82711 (12)	0.22438 (10)	0.0198 (3)	
C14	0.77051 (15)	0.94074 (12)	0.22560 (10)	0.0200 (3)	
C15	0.91203 (15)	0.99051 (12)	0.18264 (10)	0.0191 (3)	
C16	0.94484 (17)	0.69740 (13)	0.15552 (12)	0.0260 (3)	
H16A	0.9254	0.6266	0.2190	0.039*	
H16B	1.0500	0.6954	0.1303	0.039*	
H16C	0.8794	0.7002	0.1011	0.039*	
C17	0.64426 (16)	0.74435 (14)	0.25364 (12)	0.0274 (3)	
H17A	0.5524	0.7836	0.2691	0.041*	
H17B	0.6680	0.6740	0.3158	0.041*	
H17C	0.6293	0.7216	0.1951	0.041*	
C18	0.64100 (16)	1.00534 (14)	0.25750 (12)	0.0277 (3)	
H18A	0.5506	0.9593	0.2721	0.042*	
H18B	0.6239	1.0822	0.2005	0.042*	
H18C	0.6640	1.0167	0.3207	0.042*	
C19	0.94394 (17)	1.11605 (13)	0.16261 (12)	0.0269 (3)	
H19A	0.8761	1.1695	0.1112	0.040*	
H19B	1.0481	1.1321	0.1353	0.040*	
H19C	0.9283	1.1281	0.2284	0.040*	
C20	1.30357 (16)	0.81839 (14)	0.06447 (12)	0.0259 (3)	
H20A	1.2869	0.8588	−0.0105	0.039*	
H20B	1.2602	0.7404	0.0914	0.039*	
H20C	1.4116	0.8097	0.0745	0.039*	
C21	1.29702 (17)	1.05308 (14)	0.06461 (12)	0.0273 (3)	
H21A	1.4057	1.0453	0.0719	0.041*	
H21B	1.2533	1.1091	0.0938	0.041*	
H21C	1.2767	1.0816	−0.0099	0.041*	
C22	0.2846 (2)	0.51076 (15)	0.92610 (13)	0.0324 (3)	
C23	0.3928 (2)	0.42008 (15)	0.95117 (13)	0.0322 (3)	
H23	0.4577	0.4115	0.8969	0.039*	
C24	0.4076 (2)	0.34204 (15)	1.05370 (14)	0.0356 (4)	
H24	0.4823	0.2804	1.0694	0.043*	
C25	0.3142 (2)	0.35320 (16)	1.13357 (14)	0.0391 (4)	
H25	0.3242	0.2994	1.2041	0.047*	
C26	0.2062 (2)	0.44329 (17)	1.10995 (15)	0.0423 (4)	
H26	0.1418	0.4516	1.1645	0.051*	
C27	0.1913 (2)	0.52142 (16)	1.00728 (15)	0.0386 (4)	
H27	0.1165	0.5830	0.9919	0.046*	
C28	0.2695 (3)	0.5961 (2)	0.81478 (16)	0.0527 (5)	
H28A	0.1631	0.6168	0.8023	0.079*	
H28B	0.3119	0.5599	0.7669	0.079*	
H28C	0.3234	0.6673	0.8024	0.079*	
F1	0.54313 (12)	0.80934 (13)	0.59177 (11)	0.0573 (4)	
F2	0.54471 (13)	0.64004 (11)	0.58649 (13)	0.0646 (4)	
F3	0.52543 (11)	0.79295 (12)	0.44650 (8)	0.0506 (3)	
O1	0.83465 (10)	0.86990 (8)	0.45646 (7)	0.01930 (19)	
O2	0.83824 (11)	0.68098 (8)	0.45356 (8)	0.02005 (19)	
O3	0.83893 (12)	0.69747 (10)	0.62510 (8)	0.0284 (2)	
S1	0.79568 (3)	0.74753 (3)	0.51933 (2)	0.01848 (8)	
Si1	1.21202 (4)	0.90663 (3)	0.13687 (3)	0.01739 (8)	
Ti1	0.97754 (2)	0.82592 (2)	0.33331 (2)	0.01345 (6)	

### (1) [Dimethylbis(η5-tetramethylcyclopentadienyl)silane](trifluoromethanesulfonato-κ2O,O')titanium(III) toluene monosolvate Atomic displacement parameters (Å^2^)

**Table:**

	*U*^11^	*U*^22^	*U*^33^	*U*^12^	*U*^13^	*U*^23^
C1	0.0178 (6)	0.0309 (7)	0.0279 (7)	−0.0036 (5)	0.0037 (5)	−0.0109 (6)
C2	0.0109 (5)	0.0186 (6)	0.0191 (6)	−0.0004 (4)	−0.0004 (4)	−0.0086 (5)
C3	0.0128 (5)	0.0181 (6)	0.0213 (6)	0.0010 (4)	−0.0021 (4)	−0.0083 (5)
C4	0.0129 (5)	0.0188 (6)	0.0190 (6)	0.0012 (4)	−0.0041 (4)	−0.0064 (5)
C5	0.0115 (5)	0.0204 (6)	0.0182 (6)	0.0014 (4)	−0.0043 (4)	−0.0082 (5)
C6	0.0116 (5)	0.0184 (6)	0.0196 (6)	−0.0004 (4)	−0.0016 (4)	−0.0084 (5)
C7	0.0260 (7)	0.0205 (6)	0.0284 (7)	0.0028 (5)	−0.0015 (5)	−0.0127 (6)
C8	0.0238 (7)	0.0208 (6)	0.0214 (6)	0.0008 (5)	−0.0051 (5)	−0.0042 (5)
C9	0.0197 (6)	0.0276 (7)	0.0198 (6)	0.0002 (5)	−0.0034 (5)	−0.0119 (5)
C10	0.0212 (6)	0.0194 (6)	0.0248 (7)	−0.0034 (5)	0.0001 (5)	−0.0106 (5)
C11	0.0166 (6)	0.0210 (6)	0.0140 (6)	−0.0002 (5)	−0.0020 (4)	−0.0058 (5)
C12	0.0160 (6)	0.0239 (6)	0.0169 (6)	−0.0013 (5)	−0.0026 (5)	−0.0089 (5)
C13	0.0143 (6)	0.0269 (7)	0.0179 (6)	−0.0007 (5)	−0.0041 (5)	−0.0079 (5)
C14	0.0152 (6)	0.0266 (7)	0.0171 (6)	0.0044 (5)	−0.0050 (5)	−0.0074 (5)
C15	0.0175 (6)	0.0205 (6)	0.0174 (6)	0.0023 (5)	−0.0037 (5)	−0.0054 (5)
C16	0.0229 (7)	0.0299 (7)	0.0315 (8)	−0.0034 (6)	−0.0009 (6)	−0.0184 (6)
C17	0.0171 (6)	0.0390 (8)	0.0287 (7)	−0.0074 (6)	−0.0023 (5)	−0.0150 (6)
C18	0.0196 (7)	0.0350 (8)	0.0268 (7)	0.0094 (6)	−0.0032 (5)	−0.0114 (6)
C19	0.0275 (7)	0.0204 (7)	0.0305 (8)	0.0026 (5)	−0.0049 (6)	−0.0076 (6)
C20	0.0199 (6)	0.0377 (8)	0.0239 (7)	−0.0002 (6)	0.0022 (5)	−0.0168 (6)
C21	0.0260 (7)	0.0296 (7)	0.0220 (7)	−0.0081 (6)	0.0020 (5)	−0.0052 (6)
C22	0.0400 (9)	0.0310 (8)	0.0288 (8)	−0.0074 (7)	−0.0056 (7)	−0.0128 (6)
C23	0.0372 (9)	0.0336 (8)	0.0328 (8)	−0.0071 (7)	0.0022 (7)	−0.0204 (7)
C24	0.0422 (9)	0.0259 (8)	0.0416 (9)	−0.0009 (7)	−0.0066 (7)	−0.0158 (7)
C25	0.0545 (11)	0.0308 (8)	0.0285 (8)	−0.0090 (8)	−0.0004 (7)	−0.0070 (7)
C26	0.0463 (10)	0.0446 (10)	0.0372 (9)	−0.0063 (8)	0.0111 (8)	−0.0191 (8)
C27	0.0362 (9)	0.0371 (9)	0.0440 (10)	0.0015 (7)	−0.0010 (7)	−0.0181 (8)
C28	0.0716 (15)	0.0499 (12)	0.0325 (10)	−0.0025 (10)	−0.0116 (9)	−0.0103 (9)
F1	0.0236 (5)	0.0954 (10)	0.0815 (9)	0.0014 (6)	0.0060 (5)	−0.0675 (8)
F2	0.0267 (5)	0.0386 (6)	0.1027 (11)	−0.0119 (5)	0.0114 (6)	−0.0012 (7)
F3	0.0183 (5)	0.0914 (9)	0.0342 (6)	−0.0009 (5)	−0.0043 (4)	−0.0157 (6)
O1	0.0173 (4)	0.0203 (5)	0.0210 (5)	−0.0008 (3)	0.0004 (3)	−0.0090 (4)
O2	0.0180 (4)	0.0193 (4)	0.0231 (5)	−0.0024 (3)	0.0008 (4)	−0.0086 (4)
O3	0.0253 (5)	0.0373 (6)	0.0180 (5)	−0.0005 (4)	−0.0004 (4)	−0.0062 (4)
S1	0.01491 (15)	0.02177 (16)	0.01749 (15)	−0.00141 (11)	0.00086 (11)	−0.00655 (12)
Si1	0.01411 (16)	0.02167 (18)	0.01623 (17)	−0.00235 (13)	0.00148 (12)	−0.00741 (14)
Ti1	0.01059 (11)	0.01561 (11)	0.01411 (11)	−0.00023 (8)	−0.00111 (8)	−0.00574 (8)

### (1) [Dimethylbis(η5-tetramethylcyclopentadienyl)silane](trifluoromethanesulfonato-κ2O,O')titanium(III) toluene monosolvate Geometric parameters (Å, º)

**Table:**

C1—F3	1.3046 (18)	C15—C19	1.5077 (19)
C1—F2	1.3114 (18)	C15—Ti1	2.3557 (13)
C1—F1	1.3166 (19)	C16—H16A	0.9800
C1—S1	1.8384 (15)	C16—H16B	0.9800
C2—C3	1.4438 (17)	C16—H16C	0.9800
C2—C6	1.4444 (17)	C17—H17A	0.9800
C2—Si1	1.8755 (13)	C17—H17B	0.9800
C2—Ti1	2.3194 (12)	C17—H17C	0.9800
C3—C4	1.4176 (18)	C18—H18A	0.9800
C3—C7	1.5047 (18)	C18—H18B	0.9800
C3—Ti1	2.3623 (12)	C18—H18C	0.9800
C4—C5	1.4135 (18)	C19—H19A	0.9800
C4—C8	1.4999 (18)	C19—H19B	0.9800
C4—Ti1	2.4755 (12)	C19—H19C	0.9800
C5—C6	1.4187 (18)	C20—Si1	1.8692 (15)
C5—C9	1.4990 (18)	C20—H20A	0.9800
C5—Ti1	2.4727 (12)	C20—H20B	0.9800
C6—C10	1.5051 (18)	C20—H20C	0.9800
C6—Ti1	2.3615 (12)	C21—Si1	1.8677 (15)
C7—H7A	0.9800	C21—H21A	0.9800
C7—H7B	0.9800	C21—H21B	0.9800
C7—H7C	0.9800	C21—H21C	0.9800
C8—H8A	0.9800	C22—C23	1.386 (2)
C8—H8B	0.9800	C22—C27	1.393 (3)
C8—H8C	0.9800	C22—C28	1.500 (2)
C9—H9A	0.9800	C23—C24	1.381 (2)
C9—H9B	0.9800	C23—H23	0.9500
C9—H9C	0.9800	C24—C25	1.381 (3)
C10—H10A	0.9800	C24—H24	0.9500
C10—H10B	0.9800	C25—C26	1.381 (3)
C10—H10C	0.9800	C25—H25	0.9500
C11—C15	1.4408 (18)	C26—C27	1.383 (3)
C11—C12	1.4420 (18)	C26—H26	0.9500
C11—Si1	1.8747 (13)	C27—H27	0.9500
C11—Ti1	2.3250 (13)	C28—H28A	0.9800
C12—C13	1.4215 (18)	C28—H28B	0.9800
C12—C16	1.5055 (19)	C28—H28C	0.9800
C12—Ti1	2.3554 (13)	O1—S1	1.4670 (10)
C13—C14	1.412 (2)	O1—Ti1	2.2704 (10)
C13—C17	1.5039 (19)	O2—S1	1.4679 (10)
C13—Ti1	2.4674 (13)	O2—Ti1	2.2697 (9)
C14—C15	1.4239 (19)	O3—S1	1.4255 (11)
C14—C18	1.5024 (19)	S1—Ti1	2.7973 (4)
C14—Ti1	2.4716 (13)		
			
F3—C1—F2	108.35 (14)	Si1—C20—H20C	109.5
F3—C1—F1	108.26 (14)	H20A—C20—H20C	109.5
F2—C1—F1	107.77 (14)	H20B—C20—H20C	109.5
F3—C1—S1	112.84 (10)	Si1—C21—H21A	109.5
F2—C1—S1	109.98 (11)	Si1—C21—H21B	109.5
F1—C1—S1	109.49 (10)	H21A—C21—H21B	109.5
C3—C2—C6	106.27 (11)	Si1—C21—H21C	109.5
C3—C2—Si1	123.84 (9)	H21A—C21—H21C	109.5
C6—C2—Si1	124.63 (9)	H21B—C21—H21C	109.5
C3—C2—Ti1	73.67 (7)	C23—C22—C27	118.20 (16)
C6—C2—Ti1	73.62 (7)	C23—C22—C28	121.03 (17)
Si1—C2—Ti1	97.66 (5)	C27—C22—C28	120.76 (18)
C4—C3—C2	108.42 (11)	C24—C23—C22	121.07 (16)
C4—C3—C7	122.41 (12)	C24—C23—H23	119.5
C2—C3—C7	128.27 (12)	C22—C23—H23	119.5
C4—C3—Ti1	77.39 (7)	C25—C24—C23	120.28 (17)
C2—C3—Ti1	70.42 (7)	C25—C24—H24	119.9
C7—C3—Ti1	126.85 (9)	C23—C24—H24	119.9
C5—C4—C3	108.47 (11)	C24—C25—C26	119.37 (17)
C5—C4—C8	126.18 (12)	C24—C25—H25	120.3
C3—C4—C8	125.07 (12)	C26—C25—H25	120.3
C5—C4—Ti1	73.29 (7)	C25—C26—C27	120.37 (17)
C3—C4—Ti1	68.63 (7)	C25—C26—H26	119.8
C8—C4—Ti1	128.48 (9)	C27—C26—H26	119.8
C4—C5—C6	108.41 (11)	C26—C27—C22	120.70 (17)
C4—C5—C9	126.45 (12)	C26—C27—H27	119.7
C6—C5—C9	124.83 (12)	C22—C27—H27	119.7
C4—C5—Ti1	73.51 (7)	C22—C28—H28A	109.5
C6—C5—Ti1	68.69 (7)	C22—C28—H28B	109.5
C9—C5—Ti1	128.51 (9)	H28A—C28—H28B	109.5
C5—C6—C2	108.40 (11)	C22—C28—H28C	109.5
C5—C6—C10	122.12 (12)	H28A—C28—H28C	109.5
C2—C6—C10	128.60 (12)	H28B—C28—H28C	109.5
C5—C6—Ti1	77.28 (7)	S1—O1—Ti1	94.46 (5)
C2—C6—Ti1	70.44 (7)	S1—O2—Ti1	94.46 (5)
C10—C6—Ti1	126.89 (9)	O3—S1—O1	117.29 (6)
C3—C7—H7A	109.5	O3—S1—O2	117.22 (6)
C3—C7—H7B	109.5	O1—S1—O2	106.45 (6)
H7A—C7—H7B	109.5	O3—S1—C1	102.50 (7)
C3—C7—H7C	109.5	O1—S1—C1	106.02 (6)
H7A—C7—H7C	109.5	O2—S1—C1	106.19 (6)
H7B—C7—H7C	109.5	O3—S1—Ti1	128.92 (5)
C4—C8—H8A	109.5	O1—S1—Ti1	54.02 (4)
C4—C8—H8B	109.5	O2—S1—Ti1	53.99 (4)
H8A—C8—H8B	109.5	C1—S1—Ti1	128.58 (5)
C4—C8—H8C	109.5	C21—Si1—C20	101.81 (7)
H8A—C8—H8C	109.5	C21—Si1—C11	115.70 (7)
H8B—C8—H8C	109.5	C20—Si1—C11	116.30 (6)
C5—C9—H9A	109.5	C21—Si1—C2	115.90 (6)
C5—C9—H9B	109.5	C20—Si1—C2	114.92 (6)
H9A—C9—H9B	109.5	C11—Si1—C2	93.08 (6)
C5—C9—H9C	109.5	O2—Ti1—O1	62.37 (3)
H9A—C9—H9C	109.5	O2—Ti1—C2	133.35 (4)
H9B—C9—H9C	109.5	O1—Ti1—C2	133.20 (4)
C6—C10—H10A	109.5	O2—Ti1—C11	134.36 (4)
C6—C10—H10B	109.5	O1—Ti1—C11	134.60 (4)
H10A—C10—H10B	109.5	C2—Ti1—C11	71.76 (5)
C6—C10—H10C	109.5	O2—Ti1—C12	98.83 (4)
H10A—C10—H10C	109.5	O1—Ti1—C12	131.35 (4)
H10B—C10—H10C	109.5	C2—Ti1—C12	93.25 (5)
C15—C11—C12	106.30 (11)	C11—Ti1—C12	35.88 (4)
C15—C11—Si1	123.57 (10)	O2—Ti1—C15	131.78 (4)
C12—C11—Si1	124.38 (10)	O1—Ti1—C15	99.17 (4)
C15—C11—Ti1	73.24 (7)	C2—Ti1—C15	92.69 (5)
C12—C11—Ti1	73.21 (7)	C11—Ti1—C15	35.85 (5)
Si1—C11—Ti1	97.49 (5)	C12—Ti1—C15	58.64 (5)
C13—C12—C11	108.44 (12)	O2—Ti1—C6	129.96 (4)
C13—C12—C16	121.83 (12)	O1—Ti1—C6	97.72 (4)
C11—C12—C16	128.97 (12)	C2—Ti1—C6	35.93 (4)
C13—C12—Ti1	77.23 (8)	C11—Ti1—C6	93.23 (5)
C11—C12—Ti1	70.91 (7)	C12—Ti1—C6	124.59 (5)
C16—C12—Ti1	125.89 (9)	C15—Ti1—C6	94.66 (5)
C14—C13—C12	108.46 (12)	O2—Ti1—C3	97.92 (4)
C14—C13—C17	127.86 (13)	O1—Ti1—C3	130.01 (4)
C12—C13—C17	123.44 (13)	C2—Ti1—C3	35.91 (4)
C14—C13—Ti1	73.56 (7)	C11—Ti1—C3	92.98 (5)
C12—C13—Ti1	68.59 (7)	C12—Ti1—C3	95.03 (5)
C17—C13—Ti1	127.84 (9)	C15—Ti1—C3	124.00 (5)
C13—C14—C15	108.22 (12)	C6—Ti1—C3	58.57 (4)
C13—C14—C18	127.43 (13)	O2—Ti1—C13	80.42 (4)
C15—C14—C18	124.17 (13)	O1—Ti1—C13	97.20 (4)
C13—C14—Ti1	73.23 (7)	C2—Ti1—C13	126.42 (5)
C15—C14—Ti1	68.44 (7)	C11—Ti1—C13	57.90 (4)
C18—C14—Ti1	127.63 (9)	C12—Ti1—C13	34.18 (4)
C14—C15—C11	108.51 (12)	C15—Ti1—C13	56.83 (5)
C14—C15—C19	122.23 (12)	C6—Ti1—C13	149.62 (5)
C11—C15—C19	128.71 (12)	C3—Ti1—C13	125.98 (5)
C14—C15—Ti1	77.36 (8)	O2—Ti1—C14	97.62 (4)
C11—C15—Ti1	70.92 (7)	O1—Ti1—C14	80.36 (4)
C19—C15—Ti1	124.61 (9)	C2—Ti1—C14	126.03 (5)
C12—C16—H16A	109.5	C11—Ti1—C14	57.89 (4)
C12—C16—H16B	109.5	C12—Ti1—C14	56.82 (5)
H16A—C16—H16B	109.5	C15—Ti1—C14	34.20 (4)
C12—C16—H16C	109.5	C6—Ti1—C14	125.37 (5)
H16A—C16—H16C	109.5	C3—Ti1—C14	149.63 (5)
H16B—C16—H16C	109.5	C13—Ti1—C14	33.21 (5)
C13—C17—H17A	109.5	O2—Ti1—C5	95.97 (4)
C13—C17—H17B	109.5	O1—Ti1—C5	78.93 (4)
H17A—C17—H17B	109.5	C2—Ti1—C5	57.86 (4)
C13—C17—H17C	109.5	C11—Ti1—C5	126.29 (4)
H17A—C17—H17C	109.5	C12—Ti1—C5	149.71 (4)
H17B—C17—H17C	109.5	C15—Ti1—C5	125.44 (5)
C14—C18—H18A	109.5	C6—Ti1—C5	34.03 (4)
C14—C18—H18B	109.5	C3—Ti1—C5	56.68 (4)
H18A—C18—H18B	109.5	C13—Ti1—C5	175.67 (4)
C14—C18—H18C	109.5	C14—Ti1—C5	146.28 (5)
H18A—C18—H18C	109.5	O2—Ti1—C4	79.07 (4)
H18B—C18—H18C	109.5	O1—Ti1—C4	96.08 (4)
C15—C19—H19A	109.5	C2—Ti1—C4	57.80 (4)
C15—C19—H19B	109.5	C11—Ti1—C4	126.05 (4)
H19A—C19—H19B	109.5	C12—Ti1—C4	125.64 (5)
C15—C19—H19C	109.5	C15—Ti1—C4	149.15 (5)
H19A—C19—H19C	109.5	C6—Ti1—C4	56.65 (4)
H19B—C19—H19C	109.5	C3—Ti1—C4	33.98 (4)
Si1—C20—H20A	109.5	C13—Ti1—C4	146.77 (5)
Si1—C20—H20B	109.5	C14—Ti1—C4	176.02 (4)
H20A—C20—H20B	109.5	C5—Ti1—C4	33.20 (4)
			
C6—C2—C3—C4	−1.79 (14)	C12—C13—C14—Ti1	−60.01 (9)
Si1—C2—C3—C4	−157.06 (9)	C17—C13—C14—Ti1	125.51 (14)
Ti1—C2—C3—C4	−68.73 (9)	C13—C14—C15—C11	1.81 (15)
C6—C2—C3—C7	−171.04 (12)	C18—C14—C15—C11	−173.69 (12)
Si1—C2—C3—C7	33.69 (18)	Ti1—C14—C15—C11	64.63 (9)
Ti1—C2—C3—C7	122.01 (13)	C13—C14—C15—C19	174.03 (12)
C6—C2—C3—Ti1	66.95 (8)	C18—C14—C15—C19	−1.5 (2)
Si1—C2—C3—Ti1	−88.33 (9)	Ti1—C14—C15—C19	−123.15 (13)
C2—C3—C4—C5	1.26 (14)	C13—C14—C15—Ti1	−62.83 (9)
C7—C3—C4—C5	171.28 (12)	C18—C14—C15—Ti1	121.68 (13)
Ti1—C3—C4—C5	−62.86 (9)	C12—C11—C15—C14	−2.63 (14)
C2—C3—C4—C8	−173.07 (12)	Si1—C11—C15—C14	−156.81 (9)
C7—C3—C4—C8	−3.1 (2)	Ti1—C11—C15—C14	−68.90 (9)
Ti1—C3—C4—C8	122.80 (12)	C12—C11—C15—C19	−174.19 (13)
C2—C3—C4—Ti1	64.12 (8)	Si1—C11—C15—C19	31.63 (19)
C7—C3—C4—Ti1	−125.86 (12)	Ti1—C11—C15—C19	119.54 (14)
C3—C4—C5—C6	−0.22 (14)	C12—C11—C15—Ti1	66.27 (9)
C8—C4—C5—C6	174.04 (12)	Si1—C11—C15—Ti1	−87.91 (9)
Ti1—C4—C5—C6	−60.13 (8)	C27—C22—C23—C24	0.2 (2)
C3—C4—C5—C9	−174.03 (12)	C28—C22—C23—C24	179.50 (17)
C8—C4—C5—C9	0.2 (2)	C22—C23—C24—C25	0.0 (3)
Ti1—C4—C5—C9	126.06 (13)	C23—C24—C25—C26	−0.1 (3)
C3—C4—C5—Ti1	59.91 (9)	C24—C25—C26—C27	0.2 (3)
C8—C4—C5—Ti1	−125.83 (13)	C25—C26—C27—C22	0.0 (3)
C4—C5—C6—C2	−0.91 (14)	C23—C22—C27—C26	−0.2 (3)
C9—C5—C6—C2	173.02 (11)	C28—C22—C27—C26	−179.46 (19)
Ti1—C5—C6—C2	−64.11 (8)	Ti1—O1—S1—O3	119.93 (6)
C4—C5—C6—C10	−171.01 (11)	Ti1—O1—S1—O2	−13.61 (6)
C9—C5—C6—C10	2.93 (19)	Ti1—O1—S1—C1	−126.39 (6)
Ti1—C5—C6—C10	125.80 (12)	Ti1—O2—S1—O3	−119.97 (6)
C4—C5—C6—Ti1	63.19 (9)	Ti1—O2—S1—O1	13.62 (6)
C9—C5—C6—Ti1	−122.87 (12)	Ti1—O2—S1—C1	126.28 (6)
C3—C2—C6—C5	1.66 (13)	F3—C1—S1—O3	178.93 (12)
Si1—C2—C6—C5	156.68 (9)	F2—C1—S1—O3	−59.96 (14)
Ti1—C2—C6—C5	68.63 (9)	F1—C1—S1—O3	58.29 (13)
C3—C2—C6—C10	170.91 (12)	F3—C1—S1—O1	55.41 (13)
Si1—C2—C6—C10	−34.07 (18)	F2—C1—S1—O1	176.52 (12)
Ti1—C2—C6—C10	−122.11 (13)	F1—C1—S1—O1	−65.24 (13)
C3—C2—C6—Ti1	−66.98 (8)	F3—C1—S1—O2	−57.55 (13)
Si1—C2—C6—Ti1	88.05 (9)	F2—C1—S1—O2	63.56 (13)
C15—C11—C12—C13	2.49 (14)	F1—C1—S1—O2	−178.20 (11)
Si1—C11—C12—C13	156.40 (10)	F3—C1—S1—Ti1	−1.03 (15)
Ti1—C11—C12—C13	68.77 (9)	F2—C1—S1—Ti1	120.08 (11)
C15—C11—C12—C16	172.51 (13)	F1—C1—S1—Ti1	−121.67 (11)
Si1—C11—C12—C16	−33.57 (19)	C15—C11—Si1—C21	−46.12 (13)
Ti1—C11—C12—C16	−121.20 (14)	C12—C11—Si1—C21	164.31 (11)
C15—C11—C12—Ti1	−66.29 (9)	Ti1—C11—Si1—C21	−120.94 (7)
Si1—C11—C12—Ti1	87.63 (9)	C15—C11—Si1—C20	−165.54 (11)
C11—C12—C13—C14	−1.42 (15)	C12—C11—Si1—C20	44.89 (13)
C16—C12—C13—C14	−172.31 (12)	Ti1—C11—Si1—C20	119.64 (7)
Ti1—C12—C13—C14	63.17 (9)	C15—C11—Si1—C2	74.64 (11)
C11—C12—C13—C17	173.35 (12)	C12—C11—Si1—C2	−74.93 (11)
C16—C12—C13—C17	2.5 (2)	Ti1—C11—Si1—C2	−0.18 (6)
Ti1—C12—C13—C17	−122.06 (13)	C3—C2—Si1—C21	−163.79 (10)
C11—C12—C13—Ti1	−64.59 (9)	C6—C2—Si1—C21	45.42 (13)
C16—C12—C13—Ti1	124.53 (13)	Ti1—C2—Si1—C21	120.78 (7)
C12—C13—C14—C15	−0.23 (15)	C3—C2—Si1—C20	−45.33 (12)
C17—C13—C14—C15	−174.71 (13)	C6—C2—Si1—C20	163.88 (10)
Ti1—C13—C14—C15	59.78 (9)	Ti1—C2—Si1—C20	−120.76 (6)
C12—C13—C14—C18	175.07 (13)	C3—C2—Si1—C11	75.62 (11)
C17—C13—C14—C18	0.6 (2)	C6—C2—Si1—C11	−75.17 (11)
Ti1—C13—C14—C18	−124.92 (14)	Ti1—C2—Si1—C11	0.18 (6)

### (2) Chlorido[dimethylbis(η5-tetramethylcyclopentadienyl)silane](trifluoromethanesulfonato-κO)titanium(IV) Crystal data

**Table:**

[Ti(CF_3_O_3_S)(C_20_H_30_Si)Cl]	*F*(000) = 1104
*M**_r_* = 530.95	*D*_x_ = 1.514 Mg m^−^^3^
Monoclinic, *P*2_1_/*n*	Mo *K*α radiation, λ = 0.71073 Å
*a* = 10.0958 (3) Å	Cell parameters from 9538 reflections
*b* = 15.8656 (5) Å	θ = 2.7–28.9°
*c* = 14.5544 (5) Å	µ = 0.67 mm^−^^1^
β = 91.9841 (8)°	*T* = 150 K
*V* = 2329.87 (13) Å^3^	Prism, brown
*Z* = 4	0.46 × 0.39 × 0.27 mm

### (2) Chlorido[dimethylbis(η5-tetramethylcyclopentadienyl)silane](trifluoromethanesulfonato-κO)titanium(IV) Data collection

**Table:**

Bruker APEXII CCD diffractometer	5616 independent reflections
Radiation source: fine-focus sealed tube	5121 reflections with *I* > 2σ(*I*)
Detector resolution: 8.3333 pixels mm^-1^	*R*_int_ = 0.021
φ and ω scans	θ_max_ = 28.0°, θ_min_ = 1.9°
Absorption correction: multi-scan (SADABS; Bruker, 2014)	*h* = −13→13
*T*_min_ = 0.78, *T*_max_ = 0.84	*k* = −20→20
34539 measured reflections	*l* = −19→19

### (2) Chlorido[dimethylbis(η5-tetramethylcyclopentadienyl)silane](trifluoromethanesulfonato-κO)titanium(IV) Refinement

**Table:**

Refinement on *F*^2^	0 restraints
Least-squares matrix: full	Hydrogen site location: inferred from neighbouring sites
*R*[*F*^2^ > 2σ(*F*^2^)] = 0.030	H-atom parameters constrained
*wR*(*F*^2^) = 0.085	*w* = 1/[σ^2^(*F*_o_^2^) + (0.0449*P*)^2^ + 1.5546*P*] where *P* = (*F*_o_^2^ + 2*F*_c_^2^)/3
*S* = 1.04	(Δ/σ)_max_ = 0.001
5616 reflections	Δρ_max_ = 0.52 e Å^−^^3^
290 parameters	Δρ_min_ = −0.43 e Å^−^^3^

### (2) Chlorido[dimethylbis(η5-tetramethylcyclopentadienyl)silane](trifluoromethanesulfonato-κO)titanium(IV) Special details

**Table:**

Geometry. All esds (except the esd in the dihedral angle between two l.s. planes) are estimated using the full covariance matrix. The cell esds are taken into account individually in the estimation of esds in distances, angles and torsion angles; correlations between esds in cell parameters are only used when they are defined by crystal symmetry. An approximate (isotropic) treatment of cell esds is used for estimating esds involving l.s. planes.

### (2) Chlorido[dimethylbis(η5-tetramethylcyclopentadienyl)silane](trifluoromethanesulfonato-κO)titanium(IV) Fractional atomic coordinates and isotropic or equivalent isotropic displacement parameters (Å^2^)

**Table:**

	*x*	*y*	*z*	*U*_iso_*/*U*_eq_	
C1	0.87010 (16)	0.09502 (10)	0.07287 (11)	0.0215 (3)	
C2	0.93422 (16)	0.14833 (10)	0.13964 (11)	0.0242 (3)	
C3	1.01692 (16)	0.20424 (10)	0.09269 (12)	0.0237 (3)	
C4	1.00415 (15)	0.18752 (10)	−0.00264 (11)	0.0214 (3)	
C5	0.91229 (15)	0.11877 (9)	−0.01688 (10)	0.0195 (3)	
C6	0.78351 (19)	0.02185 (10)	0.09707 (12)	0.0282 (3)	
H6A	0.7986	0.0075	0.1621	0.042*	
H6B	0.8051	−0.0268	0.0590	0.042*	
H6C	0.6903	0.0371	0.0860	0.042*	
C7	0.9267 (2)	0.14231 (13)	0.24187 (12)	0.0350 (4)	
H7A	1.0121	0.1230	0.2681	0.052*	
H7B	0.8573	0.1021	0.2575	0.052*	
H7C	0.9058	0.1978	0.2670	0.052*	
C8	1.11255 (18)	0.26533 (12)	0.13645 (14)	0.0328 (4)	
H8A	1.0806	0.2826	0.1964	0.049*	
H8B	1.1202	0.3150	0.0969	0.049*	
H8C	1.1996	0.2385	0.1447	0.049*	
C9	1.09811 (17)	0.22400 (11)	−0.07006 (13)	0.0295 (4)	
H9A	1.0963	0.2857	−0.0662	0.044*	
H9B	1.0711	0.2063	−0.1325	0.044*	
H9C	1.1881	0.2038	−0.0554	0.044*	
C10	0.7318 (2)	−0.00588 (11)	−0.13260 (13)	0.0329 (4)	
H10A	0.6781	−0.0103	−0.1898	0.049*	
H10B	0.6745	−0.0116	−0.0800	0.049*	
H10C	0.7985	−0.0508	−0.1306	0.049*	
C11	0.90950 (19)	0.10131 (12)	−0.23582 (12)	0.0314 (4)	
H11A	0.9698	0.0530	−0.2372	0.047*	
H11B	0.9607	0.1537	−0.2384	0.047*	
H11C	0.8471	0.0986	−0.2888	0.047*	
C12	0.59174 (14)	0.19625 (10)	−0.05544 (10)	0.0193 (3)	
C13	0.56798 (15)	0.28149 (10)	−0.03005 (11)	0.0211 (3)	
C14	0.66644 (15)	0.33208 (10)	−0.06821 (10)	0.0202 (3)	
C15	0.75316 (15)	0.27912 (9)	−0.11656 (10)	0.0186 (3)	
C16	0.70697 (14)	0.19312 (9)	−0.11032 (9)	0.0173 (3)	
C17	0.49988 (16)	0.12536 (11)	−0.03345 (12)	0.0269 (3)	
H17A	0.4694	0.1322	0.0293	0.040*	
H17B	0.5468	0.0716	−0.0384	0.040*	
H17C	0.4235	0.1260	−0.0768	0.040*	
C18	0.45215 (17)	0.31396 (13)	0.02048 (13)	0.0324 (4)	
H18A	0.4790	0.3638	0.0563	0.049*	
H18B	0.4209	0.2702	0.0620	0.049*	
H18C	0.3806	0.3292	−0.0237	0.049*	
C19	0.67144 (19)	0.42629 (11)	−0.06534 (13)	0.0301 (4)	
H19A	0.6213	0.4492	−0.1185	0.045*	
H19B	0.7638	0.4450	−0.0670	0.045*	
H19C	0.6324	0.4463	−0.0086	0.045*	
C20	0.85573 (17)	0.31524 (11)	−0.17744 (12)	0.0273 (3)	
H20A	0.8126	0.3519	−0.2237	0.041*	
H20B	0.9017	0.2693	−0.2082	0.041*	
H20C	0.9199	0.3481	−0.1402	0.041*	
C21	0.92606 (18)	0.49005 (11)	0.14872 (13)	0.0319 (4)	
Cl1	0.64690 (4)	0.21108 (3)	0.15956 (3)	0.02798 (10)	
F1	0.89370 (14)	0.53045 (8)	0.07146 (9)	0.0494 (3)	
F2	1.05184 (12)	0.46705 (8)	0.14446 (10)	0.0484 (3)	
F3	0.91613 (13)	0.54360 (8)	0.21805 (9)	0.0500 (3)	
O1	0.84799 (11)	0.34845 (7)	0.08518 (7)	0.0206 (2)	
O2	0.86681 (16)	0.36367 (9)	0.25169 (9)	0.0399 (3)	
O3	0.68782 (13)	0.43477 (9)	0.16428 (10)	0.0370 (3)	
S1	0.81793 (4)	0.39961 (3)	0.16735 (3)	0.02400 (10)	
Si1	0.81618 (4)	0.09881 (3)	−0.12776 (3)	0.01962 (10)	
Ti1	0.78529 (2)	0.23242 (2)	0.03725 (2)	0.01467 (7)	

### (2) Chlorido[dimethylbis(η5-tetramethylcyclopentadienyl)silane](trifluoromethanesulfonato-κO)titanium(IV) Atomic displacement parameters (Å^2^)

**Table:**

	*U*^11^	*U*^22^	*U*^33^	*U*^12^	*U*^13^	*U*^23^
C1	0.0255 (7)	0.0178 (7)	0.0210 (7)	0.0011 (6)	−0.0020 (6)	0.0017 (6)
C2	0.0283 (8)	0.0229 (8)	0.0208 (7)	0.0020 (6)	−0.0070 (6)	0.0015 (6)
C3	0.0201 (7)	0.0224 (7)	0.0281 (8)	0.0012 (6)	−0.0063 (6)	−0.0019 (6)
C4	0.0176 (7)	0.0200 (7)	0.0265 (8)	0.0001 (5)	−0.0007 (6)	−0.0019 (6)
C5	0.0203 (7)	0.0164 (7)	0.0218 (7)	0.0009 (5)	0.0014 (5)	−0.0007 (5)
C6	0.0396 (9)	0.0195 (8)	0.0254 (8)	−0.0055 (7)	0.0006 (7)	0.0050 (6)
C7	0.0483 (11)	0.0367 (10)	0.0192 (8)	0.0033 (8)	−0.0104 (7)	0.0029 (7)
C8	0.0247 (8)	0.0360 (10)	0.0368 (10)	−0.0040 (7)	−0.0104 (7)	−0.0072 (8)
C9	0.0208 (7)	0.0302 (9)	0.0378 (10)	−0.0061 (6)	0.0052 (7)	−0.0008 (7)
C10	0.0438 (10)	0.0219 (8)	0.0330 (9)	−0.0092 (7)	0.0007 (8)	−0.0069 (7)
C11	0.0386 (9)	0.0323 (9)	0.0239 (8)	−0.0019 (7)	0.0101 (7)	−0.0076 (7)
C12	0.0173 (7)	0.0229 (7)	0.0175 (7)	−0.0058 (5)	−0.0020 (5)	0.0001 (5)
C13	0.0182 (7)	0.0245 (8)	0.0204 (7)	−0.0008 (6)	−0.0031 (5)	−0.0007 (6)
C14	0.0219 (7)	0.0197 (7)	0.0186 (7)	−0.0011 (5)	−0.0054 (5)	0.0017 (5)
C15	0.0215 (7)	0.0199 (7)	0.0142 (6)	−0.0053 (5)	−0.0022 (5)	0.0020 (5)
C16	0.0202 (7)	0.0193 (7)	0.0122 (6)	−0.0059 (5)	−0.0017 (5)	0.0002 (5)
C17	0.0228 (7)	0.0290 (8)	0.0290 (8)	−0.0113 (6)	0.0025 (6)	−0.0005 (7)
C18	0.0214 (8)	0.0387 (10)	0.0372 (10)	0.0027 (7)	0.0029 (7)	−0.0064 (8)
C19	0.0381 (9)	0.0190 (8)	0.0326 (9)	−0.0004 (7)	−0.0067 (7)	0.0015 (7)
C20	0.0321 (8)	0.0254 (8)	0.0245 (8)	−0.0091 (6)	0.0051 (6)	0.0057 (6)
C21	0.0349 (9)	0.0272 (9)	0.0342 (9)	−0.0127 (7)	0.0093 (7)	−0.0131 (7)
Cl1	0.0321 (2)	0.0305 (2)	0.02182 (19)	−0.00467 (16)	0.00684 (15)	0.00270 (15)
F1	0.0689 (9)	0.0324 (6)	0.0472 (7)	−0.0205 (6)	0.0045 (6)	0.0030 (5)
F2	0.0319 (6)	0.0439 (7)	0.0702 (9)	−0.0163 (5)	0.0126 (6)	−0.0186 (6)
F3	0.0599 (8)	0.0400 (7)	0.0516 (7)	−0.0251 (6)	0.0222 (6)	−0.0304 (6)
O1	0.0241 (5)	0.0198 (5)	0.0179 (5)	−0.0044 (4)	0.0014 (4)	−0.0047 (4)
O2	0.0576 (9)	0.0423 (8)	0.0195 (6)	−0.0121 (7)	−0.0013 (6)	−0.0043 (5)
O3	0.0296 (6)	0.0348 (7)	0.0473 (8)	−0.0048 (5)	0.0129 (6)	−0.0135 (6)
S1	0.0279 (2)	0.02325 (19)	0.02114 (19)	−0.00863 (14)	0.00550 (14)	−0.00723 (14)
Si1	0.0245 (2)	0.0176 (2)	0.0169 (2)	−0.00461 (15)	0.00285 (15)	−0.00384 (15)
Ti1	0.01655 (13)	0.01508 (13)	0.01236 (12)	−0.00346 (9)	0.00027 (9)	−0.00043 (9)

### (2) Chlorido[dimethylbis(η5-tetramethylcyclopentadienyl)silane](trifluoromethanesulfonato-κO)titanium(IV) Geometric parameters (Å, º)

**Table:**

C1—C2	1.427 (2)	C12—C16	1.435 (2)
C1—C5	1.438 (2)	C12—C17	1.499 (2)
C1—C6	1.502 (2)	C12—Ti1	2.4051 (14)
C1—Ti1	2.3923 (15)	C13—C14	1.407 (2)
C2—C3	1.411 (2)	C13—C18	1.495 (2)
C2—C7	1.495 (2)	C13—Ti1	2.4960 (15)
C2—Ti1	2.4711 (15)	C14—C15	1.418 (2)
C3—C4	1.414 (2)	C14—C19	1.496 (2)
C3—C8	1.495 (2)	C14—Ti1	2.4835 (15)
C3—Ti1	2.4878 (15)	C15—C16	1.446 (2)
C4—C5	1.442 (2)	C15—C20	1.500 (2)
C4—C9	1.504 (2)	C15—Ti1	2.3696 (14)
C4—Ti1	2.4116 (15)	C16—Si1	1.8810 (16)
C5—Si1	1.8809 (16)	C16—Ti1	2.3472 (14)
C5—Ti1	2.3634 (15)	C17—H17A	0.9800
C6—H6A	0.9800	C17—H17B	0.9800
C6—H6B	0.9800	C17—H17C	0.9800
C6—H6C	0.9800	C18—H18A	0.9800
C7—H7A	0.9800	C18—H18B	0.9800
C7—H7B	0.9800	C18—H18C	0.9800
C7—H7C	0.9800	C19—H19A	0.9800
C8—H8A	0.9800	C19—H19B	0.9800
C8—H8B	0.9800	C19—H19C	0.9800
C8—H8C	0.9800	C20—H20A	0.9800
C9—H9A	0.9800	C20—H20B	0.9800
C9—H9B	0.9800	C20—H20C	0.9800
C9—H9C	0.9800	C21—F2	1.325 (2)
C10—Si1	1.8670 (17)	C21—F1	1.325 (2)
C10—H10A	0.9800	C21—F3	1.325 (2)
C10—H10B	0.9800	C21—S1	1.8287 (18)
C10—H10C	0.9800	Cl1—Ti1	2.3255 (5)
C11—Si1	1.8614 (18)	O1—S1	1.4854 (11)
C11—H11A	0.9800	O1—Ti1	2.0605 (11)
C11—H11B	0.9800	O2—S1	1.4260 (14)
C11—H11C	0.9800	O3—S1	1.4265 (14)
C12—C13	1.424 (2)		
			
C2—C1—C5	108.77 (14)	C13—C18—H18B	109.5
C2—C1—C6	123.53 (14)	H18A—C18—H18B	109.5
C5—C1—C6	127.48 (14)	C13—C18—H18C	109.5
C2—C1—Ti1	75.99 (9)	H18A—C18—H18C	109.5
C5—C1—Ti1	71.30 (8)	H18B—C18—H18C	109.5
C6—C1—Ti1	123.24 (11)	C14—C19—H19A	109.5
C3—C2—C1	107.76 (14)	C14—C19—H19B	109.5
C3—C2—C7	124.98 (15)	H19A—C19—H19B	109.5
C1—C2—C7	127.04 (16)	C14—C19—H19C	109.5
C3—C2—Ti1	74.12 (9)	H19A—C19—H19C	109.5
C1—C2—Ti1	69.94 (8)	H19B—C19—H19C	109.5
C7—C2—Ti1	125.71 (12)	C15—C20—H20A	109.5
C2—C3—C4	108.78 (14)	C15—C20—H20B	109.5
C2—C3—C8	125.85 (16)	H20A—C20—H20B	109.5
C4—C3—C8	125.12 (16)	C15—C20—H20C	109.5
C2—C3—Ti1	72.82 (9)	H20A—C20—H20C	109.5
C4—C3—Ti1	70.29 (9)	H20B—C20—H20C	109.5
C8—C3—Ti1	127.42 (12)	F2—C21—F1	107.58 (16)
C3—C4—C5	108.62 (14)	F2—C21—F3	108.06 (15)
C3—C4—C9	121.99 (14)	F1—C21—F3	108.23 (16)
C5—C4—C9	127.90 (15)	F2—C21—S1	111.65 (13)
C3—C4—Ti1	76.21 (9)	F1—C21—S1	111.99 (12)
C5—C4—Ti1	70.61 (8)	F3—C21—S1	109.19 (12)
C9—C4—Ti1	130.33 (11)	S1—O1—Ti1	133.78 (7)
C1—C5—C4	106.07 (13)	O2—S1—O3	118.31 (9)
C1—C5—Si1	125.10 (11)	O2—S1—O1	113.60 (8)
C4—C5—Si1	124.11 (12)	O3—S1—O1	113.71 (7)
C1—C5—Ti1	73.50 (9)	O2—S1—C21	104.52 (9)
C4—C5—Ti1	74.26 (9)	O3—S1—C21	104.02 (9)
Si1—C5—Ti1	98.32 (6)	O1—S1—C21	99.77 (7)
C1—C6—H6A	109.5	C11—Si1—C10	103.34 (8)
C1—C6—H6B	109.5	C11—Si1—C5	117.40 (8)
H6A—C6—H6B	109.5	C10—Si1—C5	113.79 (8)
C1—C6—H6C	109.5	C11—Si1—C16	114.41 (8)
H6A—C6—H6C	109.5	C10—Si1—C16	116.35 (8)
H6B—C6—H6C	109.5	C5—Si1—C16	92.16 (6)
C2—C7—H7A	109.5	O1—Ti1—Cl1	93.24 (3)
C2—C7—H7B	109.5	O1—Ti1—C16	129.56 (5)
H7A—C7—H7B	109.5	Cl1—Ti1—C16	118.00 (4)
C2—C7—H7C	109.5	O1—Ti1—C5	129.10 (5)
H7A—C7—H7C	109.5	Cl1—Ti1—C5	119.43 (4)
H7B—C7—H7C	109.5	C16—Ti1—C5	70.23 (5)
C3—C8—H8A	109.5	O1—Ti1—C15	94.07 (5)
C3—C8—H8B	109.5	Cl1—Ti1—C15	134.62 (4)
H8A—C8—H8B	109.5	C16—Ti1—C15	35.69 (5)
C3—C8—H8C	109.5	C5—Ti1—C15	88.95 (5)
H8A—C8—H8C	109.5	O1—Ti1—C1	129.59 (5)
H8B—C8—H8C	109.5	Cl1—Ti1—C1	85.49 (4)
C4—C9—H9A	109.5	C16—Ti1—C1	93.61 (5)
C4—C9—H9B	109.5	C5—Ti1—C1	35.20 (5)
H9A—C9—H9B	109.5	C15—Ti1—C1	121.73 (5)
C4—C9—H9C	109.5	O1—Ti1—C12	129.58 (5)
H9A—C9—H9C	109.5	Cl1—Ti1—C12	84.07 (4)
H9B—C9—H9C	109.5	C16—Ti1—C12	35.12 (5)
Si1—C10—H10A	109.5	C5—Ti1—C12	94.05 (5)
Si1—C10—H10B	109.5	C15—Ti1—C12	57.50 (5)
H10A—C10—H10B	109.5	C1—Ti1—C12	100.48 (5)
Si1—C10—H10C	109.5	O1—Ti1—C4	94.14 (5)
H10A—C10—H10C	109.5	Cl1—Ti1—C4	136.14 (4)
H10B—C10—H10C	109.5	C16—Ti1—C4	89.07 (5)
Si1—C11—H11A	109.5	C5—Ti1—C4	35.14 (5)
Si1—C11—H11B	109.5	C15—Ti1—C4	87.80 (5)
H11A—C11—H11B	109.5	C1—Ti1—C4	57.25 (5)
Si1—C11—H11C	109.5	C12—Ti1—C4	121.86 (5)
H11A—C11—H11C	109.5	O1—Ti1—C2	96.02 (5)
H11B—C11—H11C	109.5	Cl1—Ti1—C2	80.15 (4)
C13—C12—C16	108.93 (13)	C16—Ti1—C2	125.95 (5)
C13—C12—C17	123.08 (14)	C5—Ti1—C2	57.54 (5)
C16—C12—C17	127.76 (14)	C15—Ti1—C2	143.04 (6)
C13—C12—Ti1	76.64 (8)	C1—Ti1—C2	34.07 (5)
C16—C12—Ti1	70.24 (8)	C12—Ti1—C2	132.45 (5)
C17—C12—Ti1	123.76 (11)	C4—Ti1—C2	56.10 (6)
C14—C13—C12	108.16 (14)	O1—Ti1—C14	77.09 (5)
C14—C13—C18	124.98 (15)	Cl1—Ti1—C14	105.99 (4)
C12—C13—C18	126.63 (15)	C16—Ti1—C14	57.55 (5)
C14—C13—Ti1	73.10 (8)	C5—Ti1—C14	122.40 (5)
C12—C13—Ti1	69.64 (8)	C15—Ti1—C14	33.87 (5)
C18—C13—Ti1	127.34 (11)	C1—Ti1—C14	151.13 (5)
C13—C14—C15	108.34 (13)	C12—Ti1—C14	55.91 (5)
C13—C14—C19	125.64 (15)	C4—Ti1—C14	117.80 (5)
C15—C14—C19	125.81 (15)	C2—Ti1—C14	170.86 (5)
C13—C14—Ti1	74.08 (9)	O1—Ti1—C3	77.08 (5)
C15—C14—Ti1	68.66 (8)	Cl1—Ti1—C3	107.97 (4)
C19—C14—Ti1	127.14 (11)	C16—Ti1—C3	122.15 (5)
C14—C15—C16	108.79 (13)	C5—Ti1—C3	57.07 (5)
C14—C15—C20	121.17 (14)	C15—Ti1—C3	117.32 (6)
C16—C15—C20	128.94 (14)	C1—Ti1—C3	55.99 (5)
C14—C15—Ti1	77.47 (9)	C12—Ti1—C3	151.09 (5)
C16—C15—Ti1	71.31 (8)	C4—Ti1—C3	33.51 (5)
C20—C15—Ti1	127.07 (11)	C2—Ti1—C3	33.06 (6)
C12—C16—C15	105.77 (13)	C14—Ti1—C3	138.03 (5)
C12—C16—Si1	126.38 (11)	O1—Ti1—C13	96.30 (5)
C15—C16—Si1	123.39 (11)	Cl1—Ti1—C13	78.61 (4)
C12—C16—Ti1	74.65 (8)	C16—Ti1—C13	57.32 (5)
C15—C16—Ti1	73.00 (8)	C5—Ti1—C13	125.93 (5)
Si1—C16—Ti1	98.88 (6)	C15—Ti1—C13	56.08 (5)
C12—C17—H17A	109.5	C1—Ti1—C13	132.25 (5)
C12—C17—H17B	109.5	C12—Ti1—C13	33.73 (5)
H17A—C17—H17B	109.5	C4—Ti1—C13	142.93 (5)
C12—C17—H17C	109.5	C2—Ti1—C13	155.96 (6)
H17A—C17—H17C	109.5	C14—Ti1—C13	32.82 (5)
H17B—C17—H17C	109.5	C3—Ti1—C13	170.73 (5)
C13—C18—H18A	109.5		
			
C5—C1—C2—C3	−0.69 (18)	C18—C13—C14—Ti1	124.13 (15)
C6—C1—C2—C3	174.19 (15)	C13—C14—C15—C16	1.11 (16)
Ti1—C1—C2—C3	−64.99 (11)	C19—C14—C15—C16	−173.83 (14)
C5—C1—C2—C7	−175.51 (16)	Ti1—C14—C15—C16	65.02 (10)
C6—C1—C2—C7	−0.6 (3)	C13—C14—C15—C20	170.13 (14)
Ti1—C1—C2—C7	120.19 (17)	C19—C14—C15—C20	−4.8 (2)
C5—C1—C2—Ti1	64.30 (11)	Ti1—C14—C15—C20	−125.97 (14)
C6—C1—C2—Ti1	−120.82 (16)	C13—C14—C15—Ti1	−63.91 (11)
C1—C2—C3—C4	0.81 (18)	C19—C14—C15—Ti1	121.15 (15)
C7—C2—C3—C4	175.76 (16)	C13—C12—C16—C15	0.61 (16)
Ti1—C2—C3—C4	−61.45 (11)	C17—C12—C16—C15	175.17 (14)
C1—C2—C3—C8	−173.60 (16)	Ti1—C12—C16—C15	−66.88 (9)
C7—C2—C3—C8	1.4 (3)	C13—C12—C16—Si1	157.29 (11)
Ti1—C2—C3—C8	124.14 (17)	C17—C12—C16—Si1	−28.1 (2)
C1—C2—C3—Ti1	62.25 (11)	Ti1—C12—C16—Si1	89.80 (10)
C7—C2—C3—Ti1	−122.79 (17)	C13—C12—C16—Ti1	67.49 (10)
C2—C3—C4—C5	−0.62 (18)	C17—C12—C16—Ti1	−117.95 (15)
C8—C3—C4—C5	173.84 (15)	C14—C15—C16—C12	−1.05 (16)
Ti1—C3—C4—C5	−63.67 (10)	C20—C15—C16—C12	−168.95 (15)
C2—C3—C4—C9	−167.72 (15)	Ti1—C15—C16—C12	68.04 (9)
C8—C3—C4—C9	6.7 (3)	C14—C15—C16—Si1	−158.61 (10)
Ti1—C3—C4—C9	129.23 (15)	C20—C15—C16—Si1	33.5 (2)
C2—C3—C4—Ti1	63.05 (11)	Ti1—C15—C16—Si1	−89.53 (10)
C8—C3—C4—Ti1	−122.49 (16)	C14—C15—C16—Ti1	−69.08 (10)
C2—C1—C5—C4	0.31 (17)	C20—C15—C16—Ti1	123.01 (16)
C6—C1—C5—C4	−174.31 (15)	Ti1—O1—S1—O2	67.83 (12)
Ti1—C1—C5—C4	67.68 (10)	Ti1—O1—S1—O3	−71.37 (12)
C2—C1—C5—Si1	−156.01 (12)	Ti1—O1—S1—C21	178.50 (10)
C6—C1—C5—Si1	29.4 (2)	F2—C21—S1—O2	58.80 (15)
Ti1—C1—C5—Si1	−88.65 (11)	F1—C21—S1—O2	179.51 (13)
C2—C1—C5—Ti1	−67.36 (11)	F3—C21—S1—O2	−60.63 (16)
C6—C1—C5—Ti1	118.01 (16)	F2—C21—S1—O3	−176.48 (13)
C3—C4—C5—C1	0.18 (17)	F1—C21—S1—O3	−55.77 (15)
C9—C4—C5—C1	166.30 (16)	F3—C21—S1—O3	64.09 (16)
Ti1—C4—C5—C1	−67.15 (10)	F2—C21—S1—O1	−58.86 (14)
C3—C4—C5—Si1	156.80 (11)	F1—C21—S1—O1	61.85 (14)
C9—C4—C5—Si1	−37.1 (2)	F3—C21—S1—O1	−178.29 (14)
Ti1—C4—C5—Si1	89.47 (11)	C1—C5—Si1—C11	−160.06 (13)
C3—C4—C5—Ti1	67.33 (11)	C4—C5—Si1—C11	47.72 (16)
C9—C4—C5—Ti1	−126.55 (16)	Ti1—C5—Si1—C11	124.30 (8)
C16—C12—C13—C14	0.06 (17)	C1—C5—Si1—C10	−39.23 (16)
C17—C12—C13—C14	−174.81 (14)	C4—C5—Si1—C10	168.55 (13)
Ti1—C12—C13—C14	63.38 (10)	Ti1—C5—Si1—C10	−114.87 (8)
C16—C12—C13—C18	174.65 (15)	C1—C5—Si1—C16	80.91 (14)
C17—C12—C13—C18	−0.2 (2)	C4—C5—Si1—C16	−71.31 (13)
Ti1—C12—C13—C18	−122.02 (16)	Ti1—C5—Si1—C16	5.27 (6)
C16—C12—C13—Ti1	−63.33 (10)	C12—C16—Si1—C11	155.74 (13)
C17—C12—C13—Ti1	121.80 (15)	C15—C16—Si1—C11	−51.39 (14)
C12—C13—C14—C15	−0.72 (17)	Ti1—C16—Si1—C11	−126.83 (8)
C18—C13—C14—C15	−175.43 (15)	C12—C16—Si1—C10	35.24 (15)
Ti1—C13—C14—C15	60.44 (10)	C15—C16—Si1—C10	−171.90 (12)
C12—C13—C14—C19	174.23 (14)	Ti1—C16—Si1—C10	112.67 (8)
C18—C13—C14—C19	−0.5 (2)	C12—C16—Si1—C5	−82.73 (13)
Ti1—C13—C14—C19	−124.61 (15)	C15—C16—Si1—C5	70.13 (13)
C12—C13—C14—Ti1	−61.16 (10)	Ti1—C16—Si1—C5	−5.31 (7)
